# HBO regulates the Warburg effect of hypoxic HCC cells through miR-103a-3p/TRIM35

**DOI:** 10.1007/s12672-024-00985-3

**Published:** 2024-04-20

**Authors:** Yuting Ding, Wenkang Luan, Zhe Wang, Bo Xie, Chengfa Sun

**Affiliations:** 1 Department of Rehabilitation, Changshu No. 2 People’s Hospital (Changshu Hospital affiliated the Nantong University), Changshu, 215500 Jiangsu China; 2 Department of Auricular Reconstruction, Plastic Surgery Hospital, Chinese Academy of Medical Sciences and Peking Union Medical College, Beijing, China; 3 School of Medicine, JiangSu University, Zhenjiang, 212013 Jiangsu China; 4 Department of Neurosurgery, Changshu No. 2 People’s Hospital (Changshu Hospital affiliated the Nantong University), Changshu, 215500 Jiangsu China

**Keywords:** Hyperbaric oxygen, miR-103a-3p, TRIM35, Warburg, Hepatocellular carcinoma

## Abstract

**Background:**

There are a lot of studies on the treatment of tumors with hyperbaric oxygen, while most of them are in breast cancer, prostate cancer and so on. However, there are still few studies on hyperbaric oxygen in treating hepatocellular carcinoma (HCC). According to the current data, hyperbaric oxygen is an effective means to intervene in tumors. The Warburg effect is a unique marker of glucose metabolism in tumors related to hypoxia, making it possible for hyperbaric oxygen to interfere with the tumor through the Warburg effect.

**Method:**

We used the hypoxia/hyperbaric oxygen(HBO)-exposed HCC cells for in vitro studies. Glucose uptake, lactic acid, and adenosine triphosphate (ATP) assessed the Warburg effect. The expression of miR-103a-3p in HCC was detected by using qRT-PCR. The effect of miR-103a-3p/TRIM35 expression level on the cells was measured using the CCK8 method and flow cytometry. The molecular biological mechanism of miR-103a-3p in HCC was examined using the luciferase reporter, MS2-RIP assays.

**Result:**

HBO inhibited the Warburg effect in hypoxic HCC cells. HBO suppressed the expression of miR-103a-3p in hypoxic HCC cells, and miR-103a-3p inhibited the expression of TRIM35 in hypoxic HCC cells. With HBO exposure, miR-103a-3p/TRIM35 regulated the Warburg effect of hypoxic HCC cells.

**Conclusion:**

These findings reveal that HBO regulates the Warburg effect of hypoxic HCC cells through miR-103a-3p/TRIM35 and inhibits tumor growth.

**Supplementary Information:**

The online version contains supplementary material available at 10.1007/s12672-024-00985-3.

## Introduction

According to the data released by the World Health Organization's 2020 Global Cancer Statistics report, there are more than 900000 new cases of liver cancer worldwide, and the global morbidity and mortality of hepatocellular carcinoma (HCC) rank fifth and third among all tumors, respectively [1, 2]. Studies have found that the vast majority of solid tumors contain areas of hypoxia [3], and hypoxia has been proven to be a contributing factor for many tumor malignant events. Hypoxia can make tumor cells more adaptable, thus promoting the progression of cancer. It has been reported that hypoxia can change the expression of some genes in tumor cells, produce various metabolic reprogramming, and induce angiogenesis to improve oxygenation [4]. This characteristic of hypoxia makes it possible for hyperbaric oxygen to treat tumors.

Hyperbaric oxygen (HBO) therapy is intermittent breathing of pure oxygen in a hyperbaric chamber at a pressure above sea level. During HBO, dissolved oxygen in plasma and oxygen-saturated hemoglobin increases oxygen availability in organs. In the aspect of independent treatment, it is reported that hyperbaric oxygen can promote the apoptosis of leukemia cells, which provides a basis for treating leukemia with hyperbaric oxygen [5]. At the same time, many studies have found that hyperbaric oxygen intervention has a significant effect on breast cancer [6, 7]. What is more, is the synergistic treatment of HBO. At present, HBO combined with radiotherapy is a more recognized way. Studies have found that HBO is an adjuvant treatment of glioma chemotherapy, which can promote neovascularization and contribute to the arrival of drugs [8]. It has also been reported that hyperbaric oxygen can regulate the tumor microenvironment, promote nano-drug delivery, and eliminate tumor stem cells in the pancreatic cancer mouse model. In Ref. [9] however, there are few studies on the treatment of liver cancer with HBO. Peng et al. have found that HBO and sorafenib alone or in combination can inhibit the progress of HCC [10]. At the same time, through the microarray analysis, it was proved that miR-103a-3p was down-regulated in human hepatocellular carcinoma cell lines treated with HBO. This study also found this feature, but whether it can become the mechanism of hyperbaric oxygen intervention against HCC has been further discussed in this paper.

Warburg effect is a unique metabolic mode in tumor cells, characterized by increased glucose uptake and lactic acid production even in aerobic environments. Different from normal cells, cancer cells tend to have glycolysis in anoxic or aerobic microenvironment [11]. Aerobic glycolysis, first found in hepatocellular carcinoma, is a marker of hepatocellular carcinoma and plays a regulatory role in proliferation, immune escape, invasion, metastasis, angiogenesis and drug resistance of hepatocellular carcinoma [12]. TRIM35 is a tumor suppressor gene [13]. Chen et al. found that TRIM35 can inhibit the Warburg effect and tumorigenicity of human hepatocellular carcinoma by binding to PKM2 [14].

This study found that hyperbaric oxygen can interfere with the Warburg effect of hepatocellular carcinoma. It can lower the level of miR-103a-3p and affect the glucose metabolism of HCC by interfering with the expression of TRIM35.

## Method and materials

### Tissue samples

Patients with liver cancer were selected from January 2023 to August 2023 in the second people's Hospital of Changshu City (Changshu Hospital affiliated to Nantong University). The exclusion criteria for this study included cases of liver cancer that were not pathologically confirmed, cases that did not provide tissue, and cases in which the sample cell mass contained less than 20% of tumor cells. The remaining cases were 20 HCC patients enrolled in the study. Each patient was pathologically diagnosed by two pathologists and provided liver cancer tissue and paracancerous normal tissue as needed. The diagnosis of cancer was initially determined by clinical and CT findings, and later confirmed by histological analysis of tumor biopsies. All participants obtained written informed consent and did not receive radiotherapy or chemotherapy before surgery. The ethical study of this study was approved by the Ethics Committee of the second people's Hospital of Changshu City (Changshu Hospital affiliated to Nantong University). All the methods in the study were conducted in accordance with the Helsinki guidelines and declarations or any other relevant guidelines. The quality and purity of all reagents are ensured by proper inspection before ordering or use, and all experiments are carried out using high-quality, standardized materials and procedures to ensure the accuracy and repeatability of the results.

### qRT-PCR

The total RNA of cell samples was extracted with Trizol reagent and reverse transcribed to cDNA, and then PCR amplification was carried out using the SYBR Premix Ex Taq kit. U6 was used as an internal reference to detect the expression of miR-103a-3p, and β-actin was used as an internal reference to detect the expression of TRIM35. The relative gene expression was calculated by the 2^−ΔΔCT^ method.

### CCK8

BEL-7402 and SK-HEP1 cells were inoculated in a 96-well plate with 1 × 10^5^cells per well. After 24 h of culture, 10 μl CCK-8 reagent (Kaiji, China) was added to each well and cultured for 2 h. An enzyme labeling instrument detected the absorbance value (OD) at 450 nm.

### Western blot

The total protein of cells in each group was cleaved and extracted with RIPA lysate. Polyacrylamide gel electrophoresis and nitrocellulose membrane transfer membrane were performed, then sealed with 5% skimmed milk powder. The antibody was added, and then exposed and developed by the gel imaging system. Antibodies against TRIM35 (Invitrogen, 1:2000, USA), PKM2 (CST,1:1000,USA), p-PKM2 (CST,1:1000,USA). β-actin (1:1000, Abcam, UK) was used for normalization.

### Flow cytometry

BEL-7402 and SK-HEP1 cells were digested and collected with pancreatic enzymes without EDTA. Add 50ul Binding Buffer to 5ul 7-AAD and mix it well. The collected cells were added to the above 7-AAD staining solution, and the reaction was carried out for 15 min under light protection. 450ul Binding Buffer was added to the mixed solution after the reaction. 1ul Annexin V-PE was added, and the reaction was carried out for 15 min against light. The apoptosis rate was detected by flow cytometry.

### Plasmid constructs and transfections

Cell culture and transfection: logarithmic BEL-7402 and SK-HEP1 cells were cultured in DMEM medium containing 10% fetal bovine serum and placed in a 37 ℃, 5% CO2 incubator. miR-103a-3p mimic, miR-103a-3p inhibitor and related negative controls were obtained from Ribobio (Guangzhou, China). The total length of TRIM35 was inserted into the eukaryotic expression vector pc DNA3.1 (Invitrogen, USA) to construct the vector. The HCC cells were transfected with miR-103a-3p mimic, inhibitor and negative control (NC) (Sangon, Shanghai, China) to up-regulate or down-regulate miR-103a-3p expression, respectively. miRNA mimic/inhibitor is a synthetic RNA molecule that can enhance or inhibit endogenous mature miRNA expression levels in cells [15].

### Lactate production, glucose uptake, and ATP levels

Lactate Assay Kit (Sigma-Aldrich, USA), Glucose Assay Kit (Sigma-Aldrich, USA), and ATP Assay Kit (Abcam, UK) were used to measure lactate, glucose, and ATP levels in blood or cells, respectively.

### Luciferase reporter assay

The TRIM35 3′ UTR containing miR-103a-3p wild-type and mutant binding sites were respectively constructed on the pmirGLO vector. The BEL-7402 and SK-HEP1 cells were cultured until a convergence degree of 70%–90% was achieved, and the miR-103a-3p mimic was co-transfected into the BEL-7402 and SK-HEP1 cells with the vector. After 48 h of culture, the dual luciferase activity was analysed using the dual luciferase reporter detection kit (Promega.USA).

### Cell culture, hypoxia and HBO exposure

BEL-7402 and SK-HEP1 were routinely cultured at 37 ℃, 10% fetal bovine serum in a 5%CO2 incubator and 1640 medium. During the hypoxia experiment, the cells were placed in the anoxic chamber of 1%O_2_ for 72 h. In the hyperbaric oxygen assay, the cultured anoxic cells BEL-7402 and SK-HEP1 were treated with HBO for 1.5 h once a day, and ensure that the oxygen concentration in the hyperbaric oxygen chamber was more than 70%, and the pressure was 2.5ATA.

### MS2-RIP(RNA binding protein immunoprecipitation) assay

TRIM35-WT and TRIM35-MUT were constructed on MS2-vectors, and the vectors were transfected into BEL-7402 and SK-HEP1 cells, respectively, and grew for 24 h. After two times of pre-cooling PBS, magnetic beads were wrapped with protein-A/G and then fully resuspended. The prepared lysates were placed in 900 μl NET2 buffer magnetic beads and kept overnight at 4℃ in a vertical mixer. After that, the sample was centrifuged to the bottom of the tube instantaneously, placed in an ice bath on a magnetic base for 1 min, and the supernatant was discarded. Add 1 ml NT2 and shake vigorously. Again, the sample was centrifuged instantaneously to the bottom of the tube and placed for 1 min. The supernatant was discarded and repeated 5 times. The sediment is the sample obtained by the RIP experiment.

## Statistical analysis

Statistical analysis was performed using GraphPad Prism version 5.0. All data are presented as mean ± standard deviation. Two independent samples were compared using the T-test. One-way analysis of variance was used for comparison among multiple groups. Result P < 0.05 meant that the difference was statistically significant. Spearman correlation analysis was analyzed by using MATLAB.

## Result

### HBO represses Warburg effect in hypoxic HCC cells

**Fig. 1:**
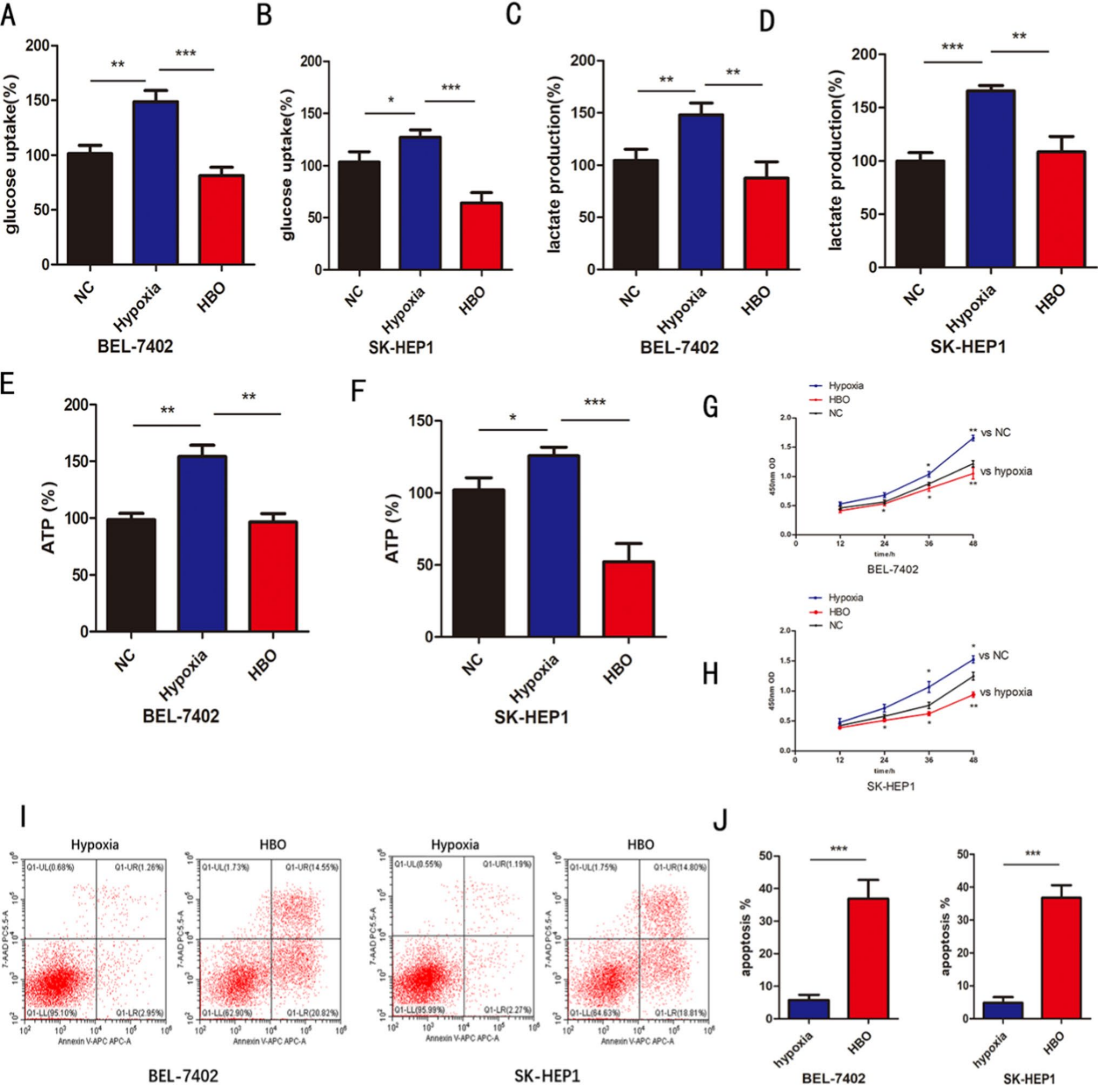
**a**, **b** Relative glucose uptake was detected in BEL-7402、SK-HEP1 cells in different group. **c**, **d** Relative lactate production was detected in BEL-7402, SK-HEP1 cells in different group. **e**, **f** Relative ATP level was detected in BEL-7402, SK-HEP1 cells in different group. **g**, **h** CCK-8 assay was used to determine the proliferation of BEL-7402、SK-HEP1 cells in each group. i-j The apoptosis in BEL-7402, SK-HEP1 cells was determined with a flow cytometer in each group. n = 3 in vitro assays, Data were expressed as the mean ± SD, *P < 0.05, **P < 0.01, ***P < 0.001.vs = versus

BEL-7402 and SK-HEP1 cells are commonly used hepatoma cell lines. We tried to treat BEL-7402 and SK-HEP1 cells with hypoxia, then put them into hyperbaric oxygen intervention, and compared the different effects. We found that glucose uptake, lactic acid production and ATP levels in BEL-7402 and SK-HEP1 cells increased under hypoxia exposure, but decreased in HBO (Fig. 1a–f). At the same time, the results of CCK8 assay and flow cytometry showed that the survival rate of BEL-7402 and SK-HEP1 cells increased and the apoptosis rate decreased under hypoxia. However, for HBO exposure, the effect is the opposite (Fig. 1g–j). Interestingly, we tried to put BEL-7402 and SK-HEP1 cells under normoxia and HBO. It was observed that HBO decreased lactic acid in BEL7402 cells, but there was no statistical significance in SK-HEP1 cells (Fig. S1). These results suggest that the state of hypoxic hepatoma cells will change after hyperbaric oxygen exposure, accompanied by the change of Warburg effect.

### Hyperbaric oxygen regulates the Warburg effect of hypoxic HCC by inhibiting the expression of miR-103a-3p

**Fig. 2:**
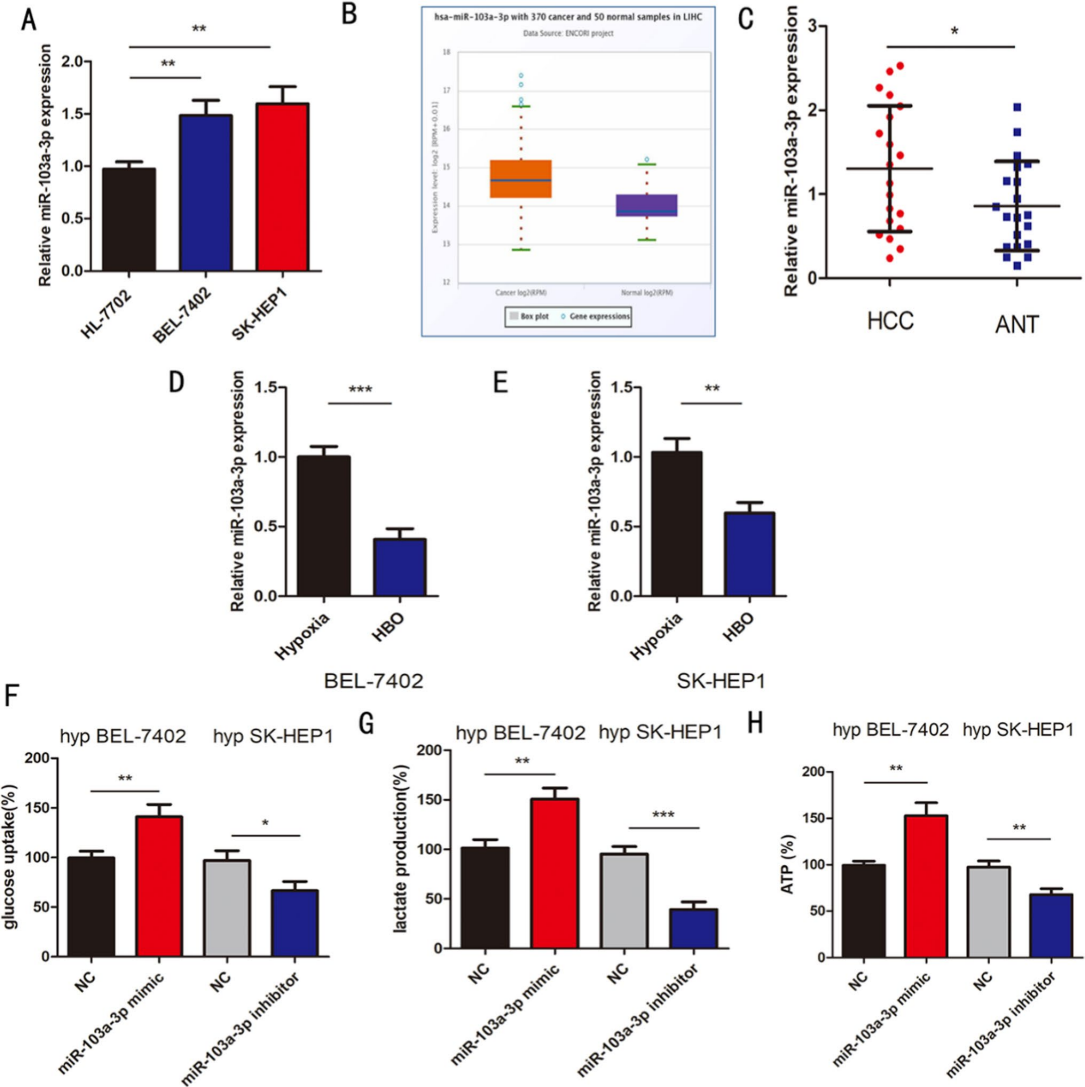
**a** qRT-PCR revealed the expression of miR-103a-3p in HL-7702、BEL-7402、SK-HEP1 cells. **b** The expression of miR-103a-3p was detected from TCGA by using ENCORI(https://rnasysu.com/encori/). **c** The miR-103a-3p expression was detected in 20 HCC tissues. **d**, **e** The expression of miR-103a-3p was detected in BEL-7402, SK-HEP1 cells between each group. **f**–**h** Relative glucose uptake, lactate production and ATP level were measured in hypoxic BEL-7402, SK-HEP1 cells with the transfection of miR-103a-3p mimic and miR-103a-3p inhibitor. n = 3 in vitro assays, Data were expressed as the mean ± SD, *P < 0.05, **P < 0.01, ***P < 0.001. hyp = hypoxia, ANT = adjacent normal tissues

Non-coding RNA is a hot topic in recent years, and it plays an important role in the regulation of tumor gene expression [16]. We speculate whether miRNA plays a role in the mechanism of warburg effect after hyperbaric oxygen exposure. We searched the literature and found that the expression of miR-103a-3p in HCC cell line decreased after hyperbaric oxygen exposure [10]. But it is not clear whether it makes sense. We used ENCORI (http://starbase.sysu.edu.cn/index.php) showed that the expression of miR-103a-3p was increased in hepatocellular carcinoma (Fig. 2b). Interestingly, the expression of miR-103a-3p in BEL7402 and SK-HEP1 human hepatocellular carcinoma cell lines was higher than that in HL-7702 normal liver cell lines (Fig. 2a). We want to know whether the expression of miR-103a-3p in hepatocellular carcinoma is significantly increased. Whether hyperbaric oxygen is related to the progression of liver cancer while inhibiting the expression of miR-103a-3p. To further verify, we collected 20 clinical samples of liver cancer tissues, consistent with the cell lines, and miR-103a-3p was up-regulated (Fig. 2c). The high expression of miR-103a-3p (expression ratio ≥ median ratio) is closely related to the clinical stage of HCC, but not to age, gender and family history (Table 1). Then, we put the hypoxic BEL-7402 and SK-HEP1 cell lines into HBO to verify the decrease of miR-103a-3p (Fig. 2d, e). We prove that miR-103a-3p is related to the Warburg effect. We explored the effects of miR-103a-3p on the Warburg of hypoxic HCC cells. Glucose uptake, lactate production, and ATP levels were decreased in hypoxic HCC cells with miR-103a-3p inhibitor and increased in hypoxic HCC cells with miR-103a-3p mimic (Fig. 2f–h).

**Table 1:** Correlation between BDNF-AS levels and clinical pathological characteristic (n = 20)

Clinical characteristics	Number	High miR-103a-3p expression	Low miR-103a-3p expression	P-value
Age				
< 50	5	1	4	0.1213
≥ 50	15	9	6	
Gender				
Male	12	7	5	0.3613
Female	8	3	5	
Family history				
Yes	10	7	3	0.0736
No	10	3	7	
TMN stage				
I-II	7	1	6	0.0191
III	13	9	4	

### MiR-103a-3p inhibits TRIM35 in hypoxic BEL-7402 and SK-HEP1 cells

**Fig. 3:**
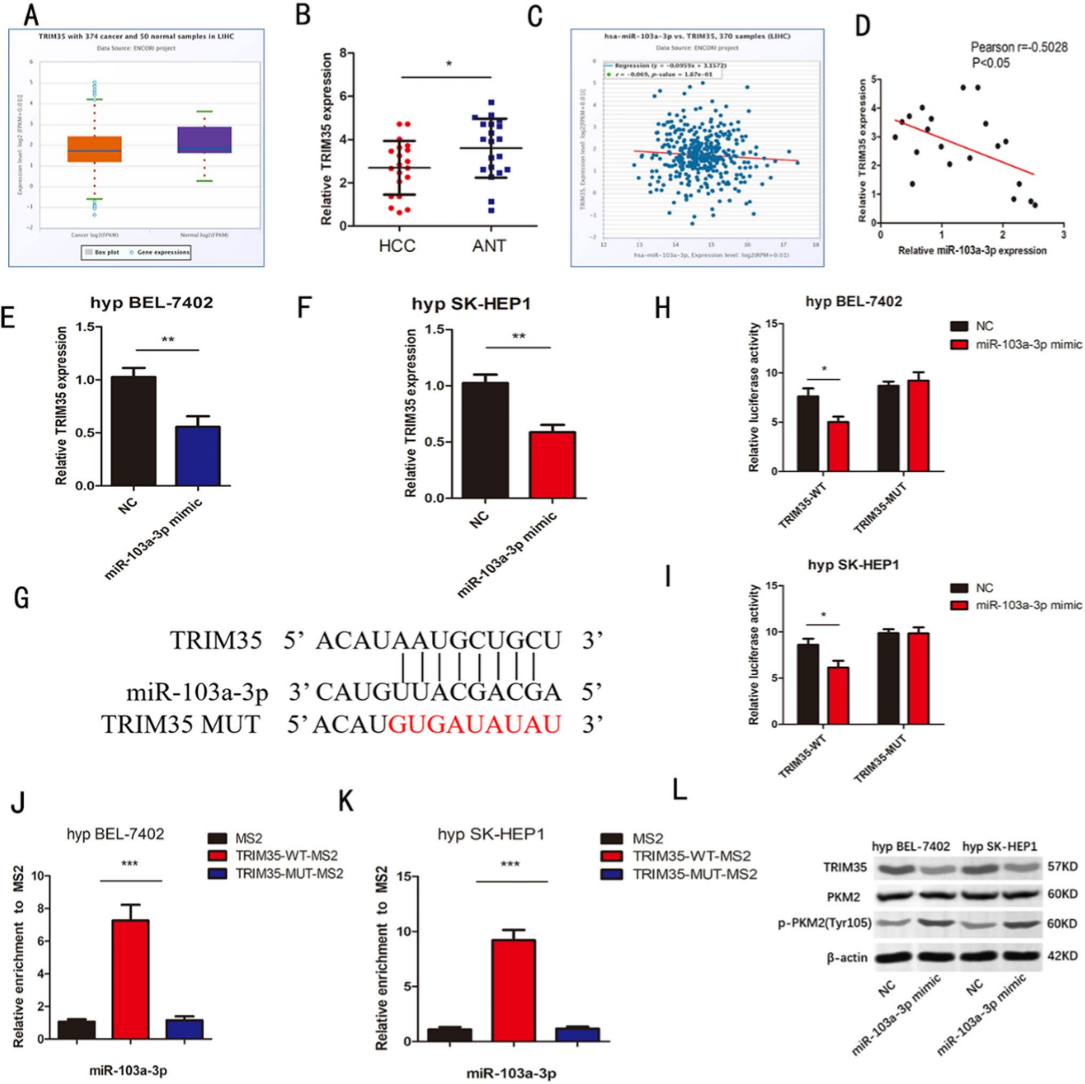
**a** The expression of TRIM35 detected from TCGA by using ENCORI(https://rnasysu.com/encori/) **b** TRIM35 expression was detected in 20 HCC tissues **c** Negative correlation between miR-103a-3p and TRIM35 expression was found in TCGA HCC dataset **d** The correlation of miR-103a-3p and TRIM35 in 20 HCC tissues was negative. **e**, **f** The levels of TRIM35 in hypoxic BEL-7402、SK-HEP1 cells following transfection with miR-103a-3p mimic or NC. g The binding sites of miR-103a-3p on TRIM35 and target sequences were mutated. h-i Relative luciferase activities of TRIM35-WT, TRIM35-MUT were analyzed in hypoxic BEL-7402、SK-HEP1 cells j-k MS2-RIP was used to detect the endogenous miR-125b-5p associated with the MS2-TRIM35 in hypoxic BEL-7402、SK-HEP1 cells l Western blots identified TRIM35、PKM2、p-PKM2(tyr105) protein expression change in different groups. n = 3 in vitro assays, Data were expressed as the mean ± SD, *P < 0.05, **P < 0.01, ***P < 0.001. hyp = hypoxia

We further explored the mechanism of miR-103a-3p regulating glucose metabolism in hypoxic hepatocellular carcinoma cells. According to previous studies, TRIM35 may have a protective effect on liver tumors [14]. The TGCA results of ENCORI showed that the expression of TRIM35 was decreased in hepatocellular carcinoma (Fig. 3a). There was negative correlation between TRIM35 and miR-103a-3p (Fig. 3c). At the same time, through the verification of clinical tissues, we also found this feature (Fig. 3b, d). It is suggested that there may be a targeted inhibition relationship between miR-103a-3p and TRIM35. MIR-103a-3p may regulate glucose metabolism through this mechanism. We transfected miR-103a-3p mimic, TRIM35 into BEL-7402 and SK-HEP1 cells. RNA expression decreased (Fig. 3e, f). In order to further study the interaction between miR-103a-3p and TRIM35, according to the ENCORI database to predict the binding site, we mutated the binding site and target sequence of miR-103a-3p in the 3 'noncoding region of TRIM35. The luciferase plasmids of wild-type and mutant TRIM35 were constructed. It was found that the luciferase activity of wild type plasmid was inhibited by miR-103a-3p, but mutant plasmid could not (Fig. 3g–i). Subsequently, we further verified the direct binding effect between miR-103a-3p and TRIM35 through MS2-RIP experiments. Compared with empty plasmids and mutants, MS2-labeled wild-type TRIM35 vectors are rich in miR-125b-5p (Fig. 3J, K). In addition, it is known that phosphorylated PKM2 of Y105 has a carcinogenic effect [17]. Our study found that miR-103a-3p inhibits the expression of TRIM35 in protein Shuiping. At the same time, the Tyr105 phosphorylation level of PKM2 was up-regulated (Fig. 3L). These results suggest that miR-103a-3p has a potential binding and inhibitory effect on TRIM35 in hypoxic HCC cells.

### With hyperbaric oxygen exposure, miR-103a-3p/ TRIM35 regulated the Warburg effect in hypoxic BEL-7402 and SK-HEP1 cells

**Fig. 4:**
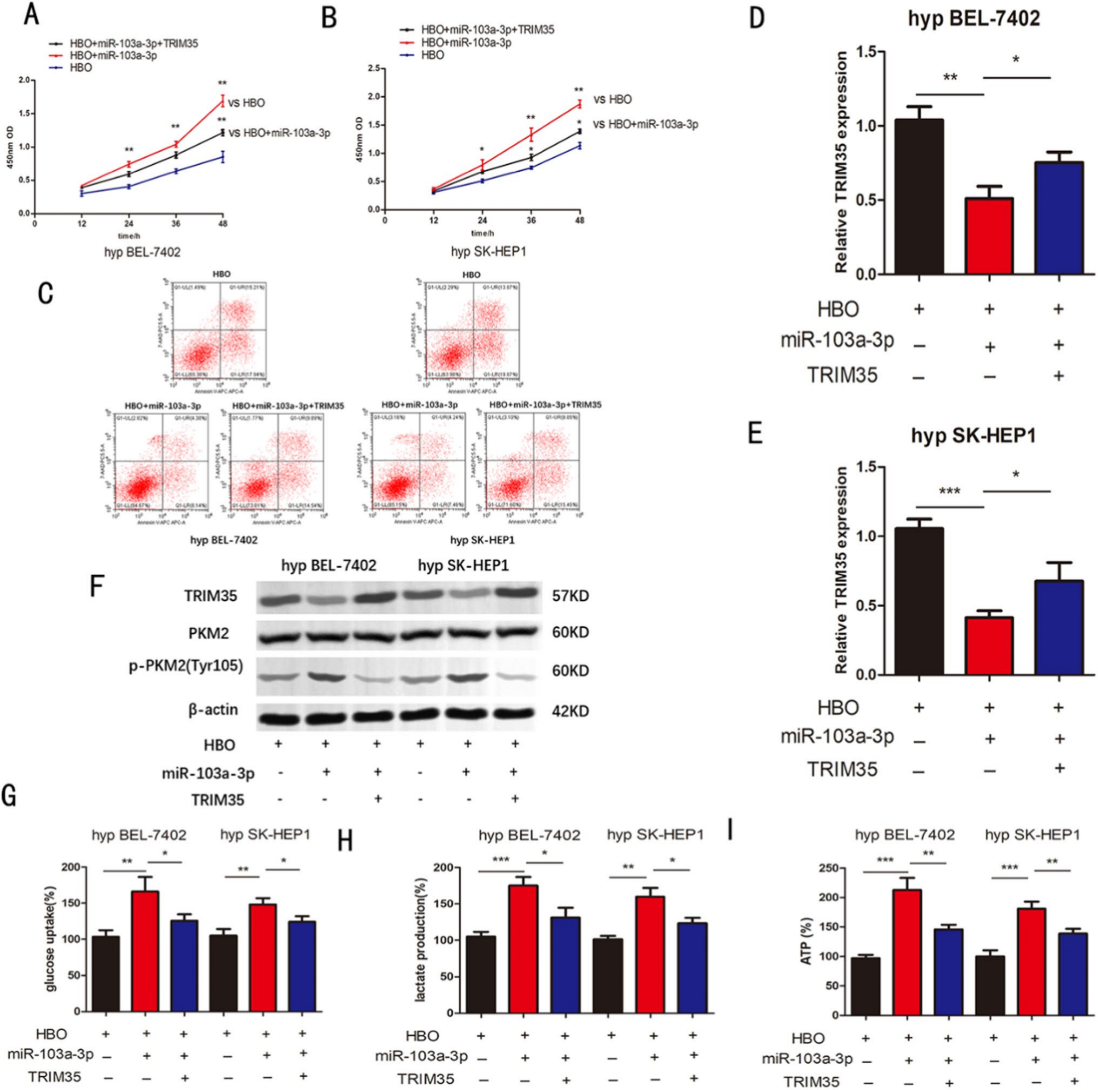
**a**, **b** The proliferative ability of model cells was determined using the CCK8 assay in different groups. c Flow cytometry was applied to detect the changes in apoptosis in each group. d-e The expression of TRIM35 mRNA in hypoxia/HBO-exposed BEL-7402, SK-HEP1 cells transfected in miR-103a-3p mimic, miR-103a-3p mimic + TRIM35 f TRIM35, PKM2, p-PKM2(Tyr105) protein expression in hypoxia/HBO-exposed BEL-7402, SK-HEP1 cells transfected with miR-103a-3p mimic, miR-103a-3p mimic + TRIM35. g-i Relative glucose uptake、lactate production, ATP levels were detected in different groups. n = 3 in vitro assays, Data were expressed as the mean ± SD, *P < 0.05, **P < 0.01, ***P < 0.001. hyp = hypoxia, vs = versus

Here, we reveal the role of miR-103a-3p/TRIM35 in regulating Warburg in BEL-7402, SK-HEP1 cells exposed to hypoxia/hyperbaric oxygen. We transfected hypoxia/HBO-exposed BEL-7402, SK-HEP1 cells with miR-103a-3p, miR-103a-3p + TRIM35. The cell viability of HBO + miR-103a-3p group was higher than that of HBO group, but the viability decreased after TRIM35 transfection (Fig. 4a, b). Flow cytometry showed that the apoptosis rate of HBO + miR-103a-3p group was lower than that of HBO group but increased after transfection of TRIM35 (Fig. 4c).Meanwhile, we also discovered that the expression of TRIM35 was down-regulated,and PKM2 phosphorylation at Tyr105 increased after transfection of miR-103a-3p, while PKM2 phosphorylation at Tyr105 decreased after co-transfection of miR-103a-3p and TRIM35 (Fig. 4d–f). In order to explore the influence of miR-103a-3p/TRIM35 on the Warburg effect of HCC cells, we detected the glucose metabolism results through a rescue experiment. Interestingly, we discovered that in HBO/hypoxic-exposed BEL-7402 and SK-HEP1 cell lines, glucose uptake, lactate production, and ATP levels in HBO + miR-103a-3p group were higher than in the HBO group. Still, the viability decreased after the transfection of TRIM35 (Fig. 4g–i). These results show that miR-103a-3p/TRIM35 modulates the Warburg effect in hypoxic HCC under HBO exposure.

## Discussion

Although there has been a lot of controversy about the relationship between hyperbaric oxygen and tumors, the evidence so far shows that hyperbaric oxygen is safe and effective in treating cancer [18]. There are frequent reports of hyperbaric oxygen alone or in combination in the treatment of tumors. The pathological mechanism of cancer is highly complex, including immune system escape, promoting tumor inflammation, activating invasion and metastasis, maintaining proliferation signal, inducing angiogenesis and metabolic reprogramming [19]. Tumor hypoxia plays a central role in many carcinogenic characteristics, which can limit the effectiveness of radiotherapy, chemotherapy and immunotherapy, thus worsening the prognosis of tumor patients [20]. In recent years, there has been more and more hyperbaric oxygen combined with radiotherapy, which the research results of chemotherapy have verified. The original treatment is more effective in improving the hypoxic area after hyperbaric oxygen intervention. Warburg effect is increasingly regarded as an essential regulator of innate and adaptive immunity and may be a marker of cancer [21]. Aerobic glycolysis is aggravated by the Warburg effect of tumor metabolic reprogramming, supporting cancer cells' rapid growth, invasion, and metastasis [22]. There are few studies on the effect of hyperbaric oxygen on the Warburg effect. Tezgin et al. reported that HBO treatment can stimulate the changes in cell energy metabolism and inhibit the Warburg effect [23]. In addition, other studies have shown that a ketogenic diet combined with hyperbaric oxygen therapy can block the Warburg effect to limit tumor growth in mice with metastatic tumors [24].

However, no similar reports have been made in HCC. In this study, in order to explore the effect of HBO on the HCC cell line, we first exposed hypoxic BEL-7402 and SK-HEP1 to hyperbaric oxygen. We observed that the specific Warburg effect of tumor cells decreased, glucose uptake, lactate production, and ATP level decreased, and the apoptosis rate decreased. Then, we further studied the mechanism. According to previous research, the miR-103a-3p of liver cancer cell lines decreased significantly with hyperbaric oxygen treatment, so miR-103a-3p became our concern. At the same time, TGCA by using ENCORI predicted that the expression of miR-103a-3p in HCC was high, and we also found the same result in the clinical evidence we collected. We also placed hypoxic BEL-7402 and SK-HEP1 in a hyperbaric oxygen environment to verify it. The decreased expression of miR-103a-3p was also observed. At the same time, we found that the Warburg effect changed after the intervention of miR-103a-3p in hypoxic BEL-7402 and SK-HEP1. Therefore, we speculate that hyperbaric oxygen affects the Warburg effect of hypoxic HCC by regulating the expression of miR-103a-3p.

TRIM35 is a relatively new tumor suppressor, which has been found to have an inhibitory effect in many kinds of tumor cells. In this paper, we verified the relationship between miR-103a-3p and TRIM35. First of all, through the bioinformatics predictions, we predict that the expression of TRIM35 in HCC is decreased and negatively correlated with the expression of miR-103a-3p. At the same time, we also verified this in the collected clinical cases of liver cancer. Then, we upregulated the expression of miR-103a-3p in HCC cell lines and found that the expression of RNA and protein of TRIM35 decreased, while that of Tyr105 phosphorylation level of PKM2 was elevated. It shows that there may be an inhibitory relationship between them. Next, Through bioinformatics search, we found their possible binding sites, and We proved that miR-103a-3p can directly bind to TRIM35 by using the luciferase reporter assay, MS2-RIP assay. More importantly, we found that in hypoxia/HBO-exposed cell lines, the cell viability and anti-apoptotic effect of miR-103a-3p could be eliminated by TRIM35, and the effect of miR-103a-3p on glycolysis was also weakened by TRIM35. Therefore, we propose that HBO regulates the targeting of miR-103a-3p to inhibit TRIM35, affecting the Warburg hypoxic HCC effect.

## Conclusion

In conclusion, these results indicated that HBO inhibited the Warburg effect, inhibited cell proliferation, and promoted apoptosis in hypoxic HCC cells. Moreover, HBO inhibited the expression of miR-103a-3p in hypoxic HCC cells, while miR-103a-3p inhibited the expression of TRIM35 in hypoxic HCC cells. In the future, HBO may play a role in the treatment of liver cancer by regulating the intervention effect of miR-103a-3p/TRIM35 on hypoxic HCC.

### Supplementary Information

Additional file1 (DOCX 89 KB)

## Data Availability

All the data and materials supporting the conclusions were included in the main paper.
