# Supplementary material for: HBO regulates the Warburg effect of hypoxic HCC cells through miR-103a-3p/TRIM35
Source: Discov Oncol. 2024 Apr 20;15:125. doi: 10.1007/s12672-024-00985-3 (PMC11032302; doi:10.1007/s12672-024-00985-3)
Supplement: Supplementary file 1 — Additional file1 (DOCX 89 KB) [file 12672_2024_985_MOESM1_ESM.docx]

**Supplementary information:**

Fig.S1

A B


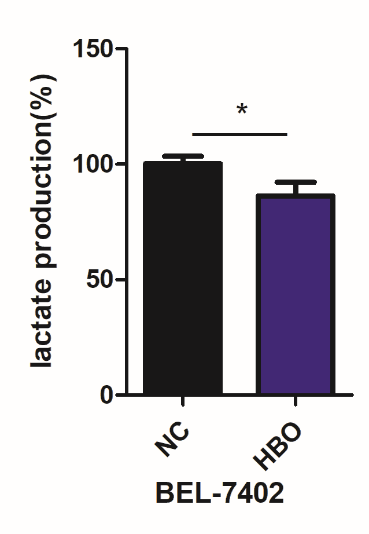

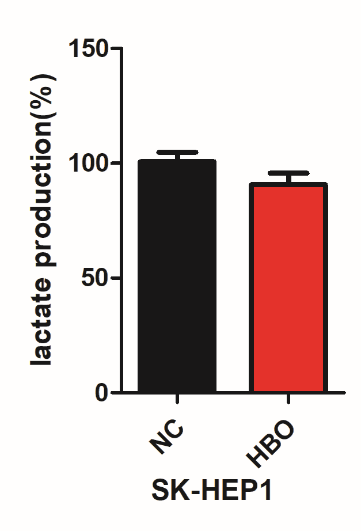


Fig.S1 a-b Relative lactate production was detected in BEL-7402、SK-HEP1 cells under normoxic condition and HBO.
